# Psychosomatic Dermatoses
*From a Lecture delivered at Bath on 26th March, 1947.


**Published:** 1947

**Authors:** W. J. O'Donovan

**Affiliations:** Physician to the Skin Department, London Hospital


### PSYCHOSOMATIC DERMATOSES*

BY

W. J. O'DONOVAN, M.D., O.B.E.

*Physician to the Skin Department, London Hospital*

---

DURING the last twenty years, attention has been given to the psychological factor in skin disease : its importance is now generally recognized. May I submit a few examples for your consideration ? Alopecia Areata was once held to be due to infection ; the idea has been dropped. It has been attributed to some obscure nervous reflex action produced by molar teeth, defects of vision, and even to nits on the eyelashes ; these ideas, too, have been abandoned. Obscure toxins from the tonsil and large bowel have been charged and absolved. But the hairdressers, nowadays called "trichologists," have always maintained that Alopecia Areata was of neurotic origin. It is found repeatedly to coincide with periods of nervous shock and strain : after a severe shock, it may even develop into total baldness.

A different type of neurosis is found in Pruritus Ani. Many causes are alleged, but the obsessional nature of these patients is worth noting. Greeting the physician with the scantiest of acknowledgment the trousers are dropped and the posterior exposed : there the patient will stand with complete satisfaction whilst one looks for physical signs to support a history that is magniloquent and logorrhæic. It is characteristic of these patients to be slow in dressing and to hang about the clinic in the hope of catching a few extra words, addressed to the students, that they may ponder over with ruminative satisfaction. They can be cured by X-ray treatment (helped naturally by sedative drugs) : even if a sheet of lead be interposed between the machine and the itching site !

The onset of Lichen Planus often corresponds with a personal calamity. I asked one patient why he remembered it had begun on the 8th and not the 5th of November, and he replied it was the day on which his eldest son had been arrested for embezzlement : you will add other histories like this to your collection. The peculiar and personal build-up of this type of patient is well illustrated by the group who suffer from Prurigo. Here we have a chronic papular irritation of the skin affecting perhaps the flexures and sometimes the whole body. We may distinguish between Prurigo mitis and

* From a Lecture delivered at Bath on 26th March, 1947.

Prurigo ferox: in the latter pitiable condition, the patient's shirt may be stuck by blood. In these patients or in their families there may be a history of asthma, had fever, colon spasm, migraine, epilepsy or (strangely) icthyosis. Two points stand out: one, that the patients are quick, clever, sensitive, emotional and artistic from their youth; and, two, that they can take extraordinary large doses of opium (e.g. 35 drops of the tincture three times daily) without hypnosis, even if the treatment be continued for a fortnight.

There is a condition of lavender-coloured, or reddish, oval, thickening of the skin of the limbs lasting for years, extremely irritable but not disabling, that has been termed Lichen Simplex Chronicus Circumscriptus. This purely descriptive term entirely hides the fact that this is mainly a nervously produced and continued skin reaction. The physician will notice its constant association with tremulous hands, restlessness, quick speech, sweating forehead and with what are called Charcot's stigmata: namely, suggestion anesthesia of the eye and soft palate to touch and of the forearm to pin-prick.

Dermatitis Artefacta, self-produced eruption, is easy to recognize because the appearance is so apart from the familiar pictures of skin diseases. Blisters occur alone in sound skin, ulcers are perfectly circular with central sloughs, sometimes arranged in line, red streaks across the limbs and the face may bear some resemblance to the tattoo-marks of the Red Indian. About these patients, the strange thing is that they think their condition is undiagnosable. I well remember, after a demonstration by a most revered senior chief, on asking the girl what the doctor thought of her, she said, "The old fool; he thinks I have done it myself." Seldom is great gain the motive for this—often some little gain in the way of compensation, interest and freedom from work. But it is worth recording that both His Majesty's Judges and Courts Martial look with profound disapproval on medical evidence that these mutilating and disfiguring lesions have been wilfully produced by the patient. Occasionally, a patient will use too much acid and it will trickle down, leaving a snail track from one edge of the ulcer and this, if photographed, may fortify the doctor to stand up to a severe cross-examination under obvious judicial disapproval.

Symmetrical red legs must be a matter of profound interest and study. These patients' troubles quite often follow a well-remembered and minor injury, remote in time, but retailed with a precision and solemnity like that of an experienced constable giving evidence in Court. Yet, in these patients, though surgical and dental wounds almost invariably heal, after the hurt the skin reddens, the leg reddens, the opposite leg shows at first a parallel scarlet scratch lines and, later, a uniform red (dry or moist) surface. It is worth noting that after several years no thrombosis, adenitis, or septicæmia develops and the locomotion of the leg is unimpaired, a condition quite unworthy of even a few months work by micro-organisms. These patients can be healed : the advertisements of patent ointments particularly appeal to them and their photographs are widely published, as photographs of patients who have fought the good fight against medical ignorance and pharmaceutical superstition and, thanks to the careful consideration of their case by the Company's directors and their fee paid in instalments, they have at last entered into the glory of cure. We, too, can cure these patients if we divorce their legs from their hands by occlusive dressings. They are given unusual treatment, such as small doses of X-rays, preferably from an old machine that has a noisy make and break attachment ; or medicine such as asafœtida, whose warmed risings in the œsophagus seem as incense to their hungry Ego.

For your last consideration, I would remind you that arsenical Exfoliative Dermatitis occurs almost solely in patients of a highly-neurotic type and secondly that, even in Psoriasis, the exacerbation can be timed not only with the Autumn and Spring changes in weather, but also with nervous upsets, particularly in the thin patient.

" A survey of the life situation and underlying emotions of patients afflicted with dermatitis and many other skin lesions draws one to the conclusion that, when a patient is obsessed with a sense of injustice, resentment and self-pity, changes in the skin sometimes take place. A time comes when irritants, such as flannel binders, spectacle frames, oil, flour, sugar or a new fur, may appear to be the last straw to break the camel's back. How partial is the importance of these things in the aetiology of the dermatitis is shown by the fact that the removal of the alleged irritant is not followed by amelioration of the dermatitis. Indeed, one surmises that if no irritant had been applied, the skin would soon have erupted spontaneously." ([1])

REFERENCE

[1] Robertson, "Emotional Aspects of Skin Disease." *Lancet*. 1947, ii.

